# Preschool instructional approaches and age 35 health and well-being

**DOI:** 10.1016/j.pmedr.2021.101498

**Published:** 2021-07-19

**Authors:** Jasmine R. Ernst, Arthur J. Reynolds

**Affiliations:** Institute of Child Development, University of Minnesota, Twin Cities, Minneapolis, MN, USA

**Keywords:** Preschool, Early childhood education, Instruction, Health, Well-being, Longitudinal

## Abstract

In this study we sought to explore the association between preschool instructional approach and health and well-being at age 35 for a large sample of low-income children. Participants included 989 low-income, minority children who attended Child-Parent Center preschools as part of the Chicago Longitudinal Study from 1983 to 1985. Preschool curriculum was obtained from teacher reports and validated by program evaluators. These data were categorized by raters as: high teacher-directed and child-initiated; low teacher-directed and child-initiated; low teacher-directed and high child-initiated; or high teacher-directed and low child-initiated. Data on adult outcomes were obtained through surveys and administrative records. Those in preschool classrooms with high teacher-directed and child-initiated instruction had increased odds of having a livable wage (Odds Ratio(OR) = 2.02, *p* = 0.001), and decreased odds of felony arrest (OR = 0.39; *p* < 0.001), jail or incarceration (OR = 0.35, *p* = 0.001), and conviction (OR = 0.52, *p* = 0.002) at age 35 than those in low teacher-directed and child-initiated classrooms. Participants experiencing low teacher-directed and high child-initiated instruction had increased odds of having a livable wage (OR = 2.01, *p* = 0.002) and decreased odds of felony arrest (OR = 0.46; *p* < 0.001), jail or incarceration (OR = 0.53; *p* = 0.023), and conviction (OR = 0.57, *p* = 0.01) at age 35. Findings were consistent across many model specifications and adjustments for potential attrition bias. Child-initiated instruction in preschool is a robust predictor of adulthood well-being. Early education prevention efforts to establish a blend of child-initiated and teacher-directed teaching philosophies affords the opportunity for long-term impacts on economic and criminal outcomes in adulthood.

## Highlights

• The association between preschool instruction and age-35 well-being was examined.
• Child-initiated instruction was consistently associated with adult well-being.
• Primarily teacher-directed instruction wasn’t a robust predictor of adult outcomes.
• Prevention efforts should blend child-initiated and teacher-directed instruction.

## 1 Introduction

High quality early educational experiences have the potential to set children on an upward trajectory (Campbell et al., 2012, Cannon et al., 2017, Reynolds and Temple, 2019, Schweinhart et al., 2005). Instructional approach is a salient aspect of educational quality because it shapes use of class time and activities relied upon for skill building. While there are multiple ways to conceptualize instructional approach, here we examined two dimensions: teacher-direction and child-initiation (e.g., Bowman et al., 2001). Teacher-directed approaches typically involve direct instruction of basic skills via structured adult-led academic lessons. Child-initiated approaches are child-led and involve allowing the child to explore and choose from a variety of options.

Typically, classrooms involve both teacher-directed and child-initiated activities but vary greatly in frequencies of these activities. Theoretical perspectives vary in which activity is thought to be most conducive to learning. Teacher-directed instruction aligns well with behaviorist theories because children learn material directly through lessons, practice skills, and receive feedback (Golbeck, 2001). Whereas child-initiated instruction aligns more with constructivist theories that posit academic and social/emotional skills are fostered through play and social interactions (Piaget, 1964, Vygotsky, 1978).

Each instructional dimension is associated with short-term positive academic and cognitive outcomes, including school readiness and literacy (Goble et al., 2016, Hayakawa and Reynolds, 2014, Graue et al., 2004). Teacher-directed approaches are associated with larger short-term academic gains (Chien et al., 2010, Goble and Pianta, 2017, Goldbeck, 2001). However, the gains from early teacher-directed approaches fade out in the primary grades, whereas the effects of high child-initiated activities remain into adolescence and young adulthood (Karnes et al., 1983, Schweinhart and Weikart, 1997, Schweinhart et al., 1986). For instance, in a predominately Black, low-income sample, children regularly exposed to teacher-directed instruction had greater felony arrests, property crimes, and misconduct at age 23 than those regularly to child-initiated instruction (Schweinhart and Weikart, 1997). Self-determination theory postulates the longevity of effects associated with child-initiated activities is likely due to the opportunity to foster autonomy, engage in enjoyable educational activities, and develop intrinsic motivation to learn (Ryan and Deci, 2000).

Exposure to more child-initiated activities might be linked to adult physical health and well-being outcomes because building a sense of autonomy, planning, making choices, and motivation might be transferable beyond immediate school contexts. These skills are important for adult outcomes, such as pursuing a higher education or making healthy choices. This is not to say teacher-directed activities aren’t essential as well. Basic skills learned through teacher-directed instruction might be foundational knowledge needed for higher-level academic tasks and/or serve as the foundation for adult daily living tasks (e.g., budgeting). Additionally, immediate feedback might foster the children’s competence in their abilities, which could extend beyond academics. Children might also rely on their teacher’s behaviors during teacher-directed instruction as a model for how to complete a task independently (e.g., reading). Teacher-directed and child-initiated activities likely work best in conjunction with one another, wherein teacher-directed activities provide the opportunity to receive immediate feedback and learn basic skills and child-initiated activities provide the opportunity to explore and foster self-regulation (Goble and Pianta, 2017, Hirsh-Pasek and Golinkoff, 2011).

A blend of high child-initiated and teacher-directed instruction was previously associated with higher educational attainment and lower criminal behavior at age 24 (Hayakawa and Reynolds, 2014). The associations between preschool instruction and well-being beyond young adulthood is unknown. Further, there is a lack of research examining if preschool instruction is associated with physical health outcomes, such as smoking or obesity. It is possible regulatory skills, planning, a sense of self-competence, and intrinsic motivation practiced in preschool activities might contribute to these specific health outcomes because theoretically these skills contribute to smoking, nutrition, and exercise.

The current study assessed the association between preschool instruction and educational attainment, income, criminal behavior, and physical health at age 35 in a predominately Black sample. We examined how preschool instructional approaches were associated with adult health and well-being beyond demographic and environmental risk factors. We hypothesized higher child-initiated and teacher-directed activities in preschool would be associated with more positive life-course outcomes in adulthood. A balance of high frequency child-initiated and teacher-directed might be ideal for yielding positive outcomes because together these practices provide adequate opportunities to foster academic skills, explore the environment, and practice regulating behaviors. Additionally, the material covered in direct instruction might be applied/extended in child-initiated activities, giving children the opportunity to learn in multiple contexts.

Although both dimensions are likely important, some constructivist theorists argue child-initiated activities are more developmentally appropriate because play and exploration provide children the opportunity to learn academic and social/emotional skills (Kamii, 1972, Vygotsky, 1978). Whereas, teacher-directed instruction focuses on academic skills, meaning children have fewer opportunities for choice and exploration. Thus, we predicted a higher frequency of child-initiated activities would be more robustly associated with adult outcomes than frequency of teacher-directed activities.

## 2 Method

### 2.1 Sample and design

The Chicago Longitudinal Study (CLS) is a prospective cohort study that began in 1985 to investigate the effects of early experiences on life-course development in a low-income, minority sample who grew up central-city Chicago (Reynolds, 2000, Reynolds et al., 2011). The original sample included 1539 participants, split among an intervention (*n* = 989) and a matched comparison (*n* = 550) group in the usual intervention. This study includes the 989 intervention children that attended Child-Parent Center (CPC) Preschools at age 3 or 4. These participants (93% Black) were born between 1979 and 1980 in the highest-poverty neighborhoods in Chicago (Reynolds et al., 2007, Reynolds and Ou, 2010). The 550 children in the CLS comparison group were not included here because the focus of this study was on the preschool instruction. Previous publications have described the sample and program (Reynolds, 1995, Reynolds, 2000).

The CPC program is a federally funded early educational intervention that provided comprehensive education and family support services from preschool to third grade (Reynolds, 1998, Reynolds and Ou, 2011). It is under the auspices of the Chicago Public School District. Key program elements included: teacher with a bachelor’s degree or higher in early childhood education, promotion of literacy skills, promotion of child-initiated and teacher-directed learning, low student-to-staff ratios, ≤17 children per classroom, parent-resource rooms to promote parent involvement, health services, and resource mobilization in the community (Reynolds, 2000, Reynolds et al., 2018). Each center is run by the Head Teacher, who acts as the director and instructional leader, and a collaborative team including the Parent-Resource Teacher and the School-Community Representative. Although the programs shared key elements, there was intentionally no uniform curriculum to allow centers to tailor the program to meet their needs. Participants attended the program for 3-hours each weekday in the preschool year. Parents of participants gave written informed consent in preschool and participants gave written informed consent at the age-35 follow-up. The study received Institutional Review Board approval.

### 2.2 Follow-up at age 35

Adult income, educational attainment, criminal behavior, and physical health data were collected as part of a larger interview at the 30-year follow-up. This interview included 130 questions about well-being, life-history, and health (https://innovation.umn.edu/cls/instruments/). Twenty-three of these questions focused on physical health. This survey was completed from August 20, 2012 to July 18, 2017 (mean age 34.9 years Reynolds et al., 2021). The majority of the participants completed this survey via telephone through the University of Minnesota and/or Northern Illinois University. Administrative data from educational, crime, and employment records complemented and took precedent over the survey in cases of disagreement.

### 2.3 Instructional approach

Head Teachers in each center (many of whom remained in their position since 1983–1985) were contacted seven years post-program to retrospectively report the frequency of each of the following activities in their center’s curriculum (all classes implemented the same approach): formal reading instruction, emphasis on basic skills, small group activities, large group activities, field trips, child-initiated activities, teacher-directed activities, and learning centers. Response options included: minimal/never, sometimes/occasionally, or often/always. Head Teachers were also asked to respond to open response questions about teaching philosophy and instructional materials/curricula. Head Teacher reports were then validated by program evaluators (Hayakawa and Reynolds, 2014, Graue et al., 2004). Trained raters used open response information on specific instructional materials and teaching philosophies to supplement the Likert responses.

**Fig. 1:**
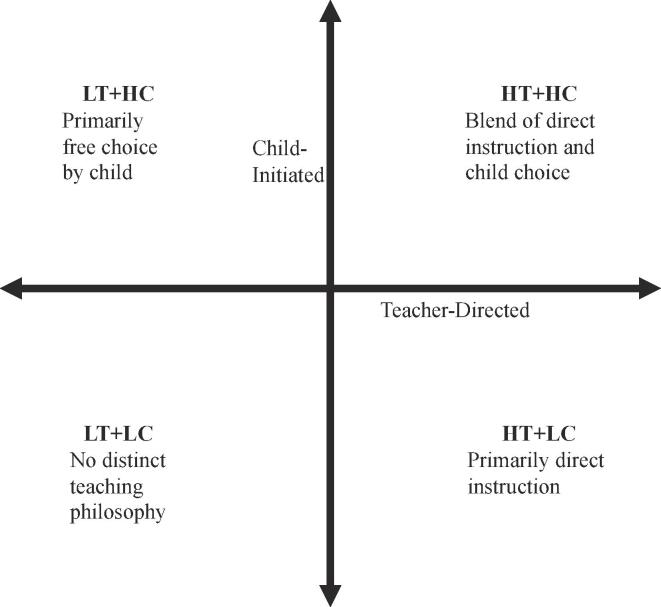
Frequency of Teacher-Directed and Child-Initiated Activities by Instructional Approach. HT + HC = High teacher-directed and high child-initiated, HT + LC = High teacher-directed and low child-initiated, LT + HC = Low teacher-directed and high child-initiated, LT + LC = Low teacher-directed and low child-initiated.

For example, if a Head Teacher mentioned using Direct Instruction System for Teaching Arithmetic and Reading, it further supported there was a high frequency of direct instruction in the classroom because this is an exemplar direct instruction program. Likert and open response curricula/teaching philosophy data were used to categorize each CPC program as high or low on two instructional dimensions in preschool: child-initiated activities and teacher-directed activities (see Fig. 1). Centers rated high on teacher-directed (HT) activities often reported higher engagement in formal reading instruction, basic skills, large-group activities, and teacher-directed activities on Likert items. Centers low in teacher-directed activities relied on activity-based curricula or materials focusing on using language in context (e.g., Peabody Language Development Kits). Centers rated high on child-initiated (HC) activities reported child-initiated activities were emphasized more frequently, as well as frequency of field trips and/or learning centers on Likert items. Table 2 shows Likert averages. Open responses items were also used to determine instruction so there were not strict Likert cut-offs for categorization. The characteristics used to determine what was considered high vs low teacher-directed and child-initiated was based on previous literature and CPC program practice (Hyson, 1991, Kamii, 1972, Reynolds, 2000, Reynolds et al., 1996).

Children in each center were assigned to one of four instructional groups based on this rating criteria: 1) high teacher-directed instruction and high child-initiated instruction (HT + HC; *n* = 387), 2) low teacher-directed instruction and high child-initiated instruction (LT + HC; *n* = 363), 3) high teacher-directed instruction and low child-initiated instruction (HT + LC; *n* = 63), or 4) low teacher-directed instruction and low child-initiated instruction (LT + LC; *n* = 176). Classrooms assigned the LT + LC instructional approach displayed no distinct teaching philosophy. Inter-rater reliability was established across three raters for categorization of each classroom into one of the four instructional categories (Cohen’s kappa = 0.75). Description of instructional variables is provided in Appendix A.

A classification of low child-initiated or teacher-directed instruction is relative rather than absolute. Classrooms categorized as low on either of these dimensions are likely to engage in similar activities as those rated higher in teacher-directed or child-initiated dimensions, but at a lower frequency.

### 2.4 Covariates

Child and family characteristics were obtained from birth records, school records, parent-report, and participant survey reports (Reynolds et al., 2001, Reynolds et al., 2007, Reynolds et al., 2009). A list and description of early childhood risk variables from ages 0–3 are in Appendix B. Descriptives of the sample used as covariates are displayed in Table 1. This sample was predominately Black, and many children experienced poverty and/or another early childhood risk factor.

**Table 1:** Descriptive Statistics for CPC Preschool Participants in the Chicago Longitudinal Study.

Covariates	CPC Preschool Group	Original Sample
	(*N* = 989)	(*N* = 1539)
Early Childhood Risk Variables		
Female child	51.80%	49.9%
Black child	92.70%	93%
Residing in high-poverty neighborhood^a^	0.446 (0.167)	0.423 (0.166)
School-level poverty	77.70%	76%
Child eligible for subsidized meals	84.20%	83.8%
Participation in Public Aid	63.10%	62.8%
Mother Did Not Completed High School	51%	54.3%
Mother Under Age 18 at Child Birth	15.60%	16.2%
Mother not Employed Full/Part Time	67.30%	66.3%
Single Parent Family Status	76.70%	76.5%
Four or more children in the household	16%	16.6%
Child Welfare Services	3.10%	3.8%
Family conflict	5.70%	5.7%
Family Financial Problems	7.10%	7%
Substance abuse parent	4.20%	4.1%
Low birthweight (<2,500 g)	10.90%	11.8%

CPC Program Variables		
Participation in CPC program beyond preschool	69.20%	55.2%
Number of years of CPC preschool^a^	1.54 (0.449)	0.99 (0.839)

*Note*. The Original Sample includes the CPC preschool group (*n* = 989) and a matched comparison group (*n* = 550) of the same age who grew up in similar neighborhoods and attended primarily randomly selected schools receiving the usual early childhood programming (Reynolds, 2000, Reynolds et al., 2011). The percentage of respective sample that received a “1″ are provided for dichotomous variables. ^a^ Means and standard deviations are provided for continuous variables.

### 2.5 Outcome measures

#### 2.5.1 Educational attainment

Two dichotomous measures of educational attainment were included: completion of an associate’s degree or higher and a bachelor’s degree or higher by age 35. Data were obtained through participant survey reports and matched with enrollment and graduation data from the National Student Clearinghouse records (Reynolds et al., 2018, National Student Clearing, 2017). The National Student Clearing house records include over 3600 public and private colleges, comprising 98% of all students.

#### 2.5.2 Income

Annual income from 2010 to 2014 was reported by participants in the age 30–35 survey (range: $0-$120,454.28, *M*=$20,994.23, *SD*=$19,630.86). Annual income was dichotomized at the livable wage ($15,592 or higher) due to the right skew of the distribution.

#### 2.5.3 Criminal behavior

Three dichotomous measures of criminal behavior were included: felony arrest, conviction, and jail or incarceration. Data were obtained through the age 30–35 survey and the Illinois Department of Employment Security records.

#### 2.5.4 Physical health

##### 2.5.4.1 Current smoking

In the age 30–35 survey, participants were asked about their history and current use of cigarettes and other tobacco products. Current smoking status was dichotomized. A positive response to the question, “How often do you currently use it (tobacco products)?” received a 1. Remaining individuals were coded 0.

##### 2.5.4.2 BMI

In the age 30–35 survey, participants reported their height and weight without shoes. Body mass index (BMI) was calculated as height (in meters) divided by weight (in kilograms) squared, and estimated body fat. According to the World Health Organization, BMI ≥ 30 is considered obese (World Health Organization, 2018). A dichotomous variable for obesity was included in our analyses.

#### 2.5.5 Statistical analyses

Analyses were conducted using IBM SPSS Statistics, Version 25, and the R software. Imputation processes were conducted for a few covariate and outcome variables; missing data ranged from 0 to 24%, and the imputation process primarily involved multiple imputation via Expectation Maximization (EM) algorithm in LISREL. Hierarchical logistical regressions were conducted to examine the associations between instructional approach and each adult well-being outcome of interest. Log-odds ratios (OR) are reported for two models, the first model included preschool instructional approach categories. The second included demographic and risk covariates. 95% confidence intervals (CIs) were calculated and reported. Participants were nested in 20 schools; however, multilevel models were unnecessary, as intraclass correlations by preschool were low (<0.03).

## 3 Results

Table 3 shows prevalence of adult outcomes by preschool instructional approach. Differences in prevalence rates by group vary by outcome, with the widest prevalence range being between LT + LC and HT + HC for adult felony arrests by age 35 (see Appendix C for correlations). It is important to note approximately 39% of the CPC program participants were in a HT + HC classroom, 37% in HT + LC, 18% in LT + LC, and 6% in HT + LC. LT + LC is the reference group in analyses.

**Table 2:** Head Teacher Reports of Frequency of Activities in the Child-Parent Centers.

Instructional Approach	How often did your curriculum contain the following?	Minimal/Never	Sometimes/Occasionally	Often/Always
HT + HC	Formal reading instruction	137 (35%)	149 (39%)	101 (26%)
	Emphasis on basic skills	0 (0%)	140 (36%)	247 (64%)
	Small group activities	0 (0%)	101 (26%)	286 (74%)
	Large group activities	0 (0%)	77 (20%)	310 (80%)
	Field trips	0 (0%)	0 (0%)	387 (100%)
	Child-initiated activities	0 (0%)	225 (58%)	162 (42%)
	Teacher-directed activities	0 (0%)	77 (20%)	310 (80%)
	Learning centers	0 (0%)	152 (39%)	235 (61%)

LT + HC	Formal reading instruction	184 (51%)	70 (19%)	109 (30%)
	Emphasis on basic skills	41 (11%)	127 (35%)	195 (54%)
	Small group activities	0 (0%)	1 (0.3%)	362 (99.7%)
	Large group activities	0 (0%)	50 (14%)	313 (86%)
	Field trips	0 (0%)	0 (0%)	363 (100%)
	Child-initiated activities	1 (0.3%)	277 (76%)	85 (23%)
	Teacher-directed activities	0 (0%)	68 (19%)	295 (81%)
	Learning centers	1 (0.3%)	36 (9.9%)	326 (90%)

HT + LC	Formal reading instruction	0 (0%)	0 (0%)	63 (100%)
	Emphasis on basic skills	0 (0%)	0 (0%)	63 (100%)
	Small group activities	0 (0%)	0 (0%)	63 (100%)
	Large group activities	0 (0%)	63 (100%)	0 (0%)
	Field trips	0 (0%)	0 (0%)	63 (100%)
	Child-initiated activities	25 (40%)	38 (60%)	0 (0%)
	Teacher-directed activities	0 (0%)	0 (0%)	63 (100%)
	Learning centers	38 (60%)	0 (0%)	25 (40%)

LT + LC	Formal reading instruction	27 (15%)	1 (0.6%)	148 (84%)
	Emphasis on basic skills	0 (0%)	27 (15%)	149 (85%)
	Small group activities	0 (0%)	175 (99%)	1 (0.6%)
	Large group activities	0 (0%)	0 (0%)	176 (100%)
	Field trips	0 (0%)	122 (69%)	54 (31%)
	Child-initiated activities	131 (74%)	45 (26%)	0 (0%)
	Teacher-directed activities	0 (0%)	0 (0%)	176 (100%)
	Learning centers	131 (74%)	45 (26%)	0 (0%)

*Note.* Likert items were only one part of instructional categorization. Coders also used open response items to determine the instructional approach category that was most appropriate. Some activities do not add up to 100% due to rounding.

**Table 3:** Prevalence of Age 35 Well-Being Outcomes by Instructional Group.

Outcomes	HT + HC (*n* = 387)	HT + LC (*n* = 63)	LT + HC (*n* = 363)	LT + LC (*n* = 176)
	*n*, Percent	*n*, Percent	*n*, Percent	*n*, Percent
Livable Wage	182, 47%	24, 38.1%	176, 48.5%	58, 33%
Associate’s degree	73, 18.9%	6, 9.5%	67, 18.5%	30, 17%
Bachelor’s degree	60, 15.5%	4, 6.3%	42, 11.6%	18, 10.2%
Felony Arrests	117, 30.2%	21, 33.3%	116, 32%	84, 47.7%
Jail or incarceration	54, 14%	16, 25.4%	62, 17.1%	44, 25%
Conviction	144, 37.2	22, 34.9%	133, 36.6%	90, 51.1%
Smoking	119, 30.7%	17, 27%	99, 27.3%	46, 26.1%
Obesity (BMI ≥ 30)	129, 33.3%	19, 30.2%	113, 31.1%	50, 28.4%

HT + HC = High teacher-directed and high child-initiated, HT + LC = High teacher-directed and low child-initiated, LT + HC = Low teacher-directed and high child-initiated, LT + LC = Low teacher-directed and low child-initiated. The *N* and percentage of the CPC program sample that received a “1″ and are provided for dichotomous variables. Some variables have missing data, see Appendix B for more information.

### 3.1 Instructional approach and adult health and well-being

Table 4 shows the associations between preschool instructional approaches and age 35 health and well-being outcome variables before (Model 1) and after (Model 2) adjusting for early childhood demographic and risk variables.

**Table 4:** Abbreviated Logistic Hierarchical Regression Odds Ratio and Confidence Intervals for Models Predicting Age 35 Outcomes.

Model & Instruction	Livable Wage	AA	BA	Felony Arrests	Jail or incarceration	Conviction	Smoking	Obesity (BMI ≥ 30)
Model 1 (unadjusted)								
HT + HC	1.936** (1.302, 2.878)	1.053 (0.65, 1.706)	1.463 (0.829, 2.582)	0.456** (0.313, 0.664)	0.481** (0.304, 0.76)	0.543** (0.376, 0.786)	1.067 (0.683, 1.668)	1.23 (0.79, 1.914)
HT + LC	1.221 (0.656, 2.272)	0.471 (0.185, 1.201)	0.532 (0.172, 1.642)	0.524* (0.283, 0.968)	0.978 (0.496, 1.93)	0.489* (0.266, 0.898)	0.795 (0.398, 1.592)	0.773 (0.389, 1.537)
LT + HC	2.146** (1.437, 3.205)	1.082 (0.663, 1.766)	1.103 (0.609, 1.997)	0.519* (0.356, 0.756)	0.64* (0.41, 0.999)	0.565** (0.389, 0.819)	0.967 (0.612, 1.527)	1.106 (0.705, 1.736)
Constant	0.596** (0.428, 0.829)	0.236** (0.157, 0.353)	0.134** (0.082, 0.22)	0.931 (0.688, 1.26)	0.333** (0.235, 0.473)	1.074 (0.794, 1.454)	0.629* (0.431, 0.916)	0.727 (0.501, 1.055)
−2 Log Likelihood	1137.5	846.34	688.39	1197.4	876.13	1256.98	919.09	922.49
Nagelkerke *R^2^*	0.027	0.008	0.012	0.026	0.02	0.018	0.002	0.005

Model 2 (adjusted)								
HT + HC	2.019** (1.315, 3.099)	1.278 (0.755, 2.165)	1.879* (1.011, 3.49)	0.392** (0.255, 0.601)	0.346** (0.198, 0.603)	0.519** (0.341, 0.791)	0.945 (0.581, 1.54)	1.184 (0.741, 1.891)
HT + LC	1.367 (0.717, 2.607)	0.5 (0.19, 1.315)	0.63 (0.197, 2.01)	0.573 (0.294, 1.115)	1.482 (0.65, 3.377)	0.475* (0.243, 0.927)	0.784 (0.377, 1.631)	0.801 (0.397, 1.615)
LT + HC	2.009** (1.305, 3.093)	1.181 (0.695, 2.006)	1.157 (0.614, 2.179)	0.46** (0.299, 0.708)	0.529* (0.306, 0.916)	0.569** (0.372, 0.872)	1.017 (0.615, 1.682)	1.054 (0.653, 1.703)
Female child	1.288 (0.964, 1.72)	2.997** (2.023, 4.442)	2.388** (1.531, 3.723)	0.213** (0.157, 0.289)	0.045** (0.024, 0.083)	0.201** (0.15, 0.27)	0.357** (0.256, 0.499)	1.252 (0.909, 1.724)
Black child	0.757 (0.413, 1.388)	0.607 (0.304, 1.211)	0.56 (0.257, 1.221)	1.208 (0.645, 2.262)	1.51 (0.635, 3.587)	1.91* (1.013, 3.6)	1.776 (0.828, 3.81)	0.937 (0.478, 1.835)
School-level poverty	0.927 (0.647, 1.328)	1.905* (1.159, 3.128)	1.934* (1.096, 3.414)	0.736 (0.508, 1.067)	0.693 (0.429, 1.121)	0.984 (0.684, 1.416)	0.969 (0.644, 1.457)	0.669* (0.45, 0.993)
Residing in high-poverty neighborhood	0.623 (0.253, 1.53)	0.6 (0.198, 1.813)	0.368 (0.105, 1.293)	1.634 (0.646, 4.134)	3.689* (1.09, 12.481)	0.981 (0.399, 2.413)	1.622 (0.576, 4.567)	0.562 (0.208, 1.524)
Child eligible for subsidized meals	0.863 (0.566, 1.315)	0.767 (0.463, 1.271)	0.79 (0.452, 1.379)	1.073 (0.688, 1.674)	1.327 (0.744, 2.368)	0.924 (0.6, 1.425)	0.998 (0.62, 1.604)	0.961 (0.611, 1.514)
Low birthweight (<2,500 g)	0.86 (0.544, 1.36)	0.792 (0.432, 1.452)	0.964 (0.483, 1.926)	1.008 (0.63, 1.612)	1.022 (0.544, 1.921)	1.041 (0.662, 1.638)	0.748 (0.447, 1.252)	0.811 (0.497, 1.323)
Number of years of CPC preschool	0.873 (0.653, 1.169)	0.771 (0.533, 1.114)	0.797 (0.521, 1.219)	0.811 (0.602, 1.094)	0.74 (0.5, 1.095)	0.942 (0.703, 1.262)	0.95 (0.682, 1.323)	1.213 (0.882, 1.668)
Missing Risk Variable	1.259 (0.777, 2.038)	1.334 (0.737, 2.411)	1.461 (0.745, 2.864)	0.603 (0.363, 1.003)	1.235 (0.671, 2.272)	0.594* (0.365, 0.967)	1.081 (0.627, 1.866)	0.771 (0.448, 1.327)
Constant	2.403 (0.895, 6.455)	0.404 (0.122, 1.337)	0.299 (0.076, 1.166)	1.838 (0.661, 5.115)	0.42 (0.106, 1.655)	1.12 (0.408, 3.073)	0.398 (0.121, 1.302)	0.992 (0.329, 2.99)
−2 Log Likelihood	1091.1	770.59	620.38	1066.5	664.05	1108.6	857.34	901.8
Nagelkerke *R^2^*	0.098	0.141	0.148	0.201	0.346	0.212	0.118	0.045
*N*	833	869	869	945	945	945	690	673

*Note:* **p < 0.01; *p < 0.05. This is an abbreviated table, such that not all covariates are shown. Full table with all covariates is available in Appendix D. HT + HC = High teacher-directed and high child-initiated, HT + LC = High teacher-directed and low child-initiated, LT + HC = Low teacher-directed and high child-initiated, LT + LC = Low teacher-directed and low child-initiated.

#### 3.1.1 Educational attainment

Preschool instructional approaches were not associated with completion of an associate’s, before or after accounting for covariates in the model. Relative to the LT + LC group, those in the HT + HC group had marginally increased odds of obtaining a bachelor’s degree (OR = 1.88; *p* = 0.046) by age 35, after controlling for covariates.

#### 3.1.2 Criminal behavior

Relative to the LT + LC group, those in the HT + HC group had a decreased odds of adult felony arrest (OR = 0.39; *p* < 0.001), jail or incarceration (OR = 0.35; *p* = 0.001), and conviction (OR = 0.52; *p* = 0.002) by age 35, after controlling for demographic and risk covariates. Similarly, those in the LT + HC group had a decreased odds of adult felony arrest (OR = 0.46; *p* < 0.001), jail or incarceration (OR = 0.53; *p* = 0.023), and conviction (OR = 0.57; *p* = 0.01) in adulthood compared to those in the LT + LC classrooms. Those in the HT + LC preschool classrooms were less likely to be convicted of a crime (OR = 0.48; *p* = 0.03) than those in LT + LC classrooms.

#### 3.1.3 Income

Relative to the LT + LC group, the HT + HC (OR = 2.02; *p* = 0.001) and LT + HC (OR = 2.01; *p* = 0.002) groups had increased odds of having an annual income at or above the livable wage at age 35, after controlling for covariates.

#### 3.1.4 Physical health

Preschool instructional approaches were not associated with current smoking or obesity (BMI ≥ 30) at age 35, before or after accounting for covariates.

### 3.2 Robustness analyses

We added inverse propensity weights to the model using risk factors to predict the probability of having a valid response on the outcome variable of interest (Appendices E-G). Due to the completeness of the criminal behavior data, we did not need to run propensity score analyses for these outcomes. The patterns of findings in robustness analyses did not differ for associate’s degree, income, or physical health, which supports the robustness of these findings. However, the finding that those in the HT + HC group were more likely to hold a bachelor’s degree by age 35 than the LT + LC group was not significant in robustness analyses. The marginally significant *p*-value in the main analyses and nonsignificant robustness tests suggest this finding should be interpreted cautiously.

Another robustness analysis examined if the effect of the HT + HC curriculum on age 35 outcomes varied for males versus females. We conducted this test because prior studies with this sample found males and females varied in their life outcomes, but found no evidence of gender moderating the association between HT + HC and adult outcomes (Eales et al., 2020, Ou and Reynolds, 2006).

## 4 Discussion

Higher frequency of child-initiated instruction in preschool was associated with indicators of well-being in adulthood, including income, felony arrest, and jail or incarceration. This pattern of findings emerged regardless of the frequency of teacher-directed activities, suggesting the child-initiated activity frequency might be driving these associations. Additionally, those in classrooms with lower frequency of child-initiated and higher frequency of teacher-directed activities were less likely to be convicted by age 35, than those in classrooms with no distinct teaching philosophy (LT + LC). These findings are consistent with prior work, suggesting child-initiated activities play a unique role in promoting well-being in adulthood (Hayakawa and Reynolds, 2014, Schweinhart and Weikart, 1997, Graue et al., 2004).

Prior studies examined the association between preschool instruction and immediate, juvenile, and adult outcomes through the mid-20s (Reynolds, 2000, Graue et al., 2004). This is one of the few studies that has examined these associations beyond early adulthood. Although causation cannot be inferred, this study contributes to the broader body of literature aiming to identify the key components of high-quality early education.

Child-directed instructional approaches were consistently associated with income and criminal behavior at age 35, but educational attainment and physical health outcomes were not. Prior work conducted up until age 24, identified an association between high child-initiated and teacher-directed activities and completion of a bachelor’s degree (Graue et al., 2004). This finding was partially replicated in the main analyses at age-35, but not robustness tests. Therefore, the association between high frequency of child-initiated and teacher-directed activities and completion of a bachelor’s degree should be interpreted with caution. It is possible we were unable to detect a robust association between these variables 10 years later due to the nature of this high-risk sample. However, the magnitude of these associations might decay over time regardless of the sample. To our knowledge, no prior work has looked at the association between preschool instruction and physical health. We selected current smoking and BMI as our physical health outcomes because they both involve aspects of self-regulation (Bub et al., 2016, deBlois and Kubzansky, 2016, Thamotharan et al., 2013) and child-initiated instructional practices have been linked to improvement in regulatory skills (Goble and Pianta, 2017). It is possible these variables are not significantly associated with one another or that this indirect association is small in magnitude. Given the health-disparities faced by low-income, Black families, more work examining links to physical health is imperative. In addition, future work could look into the role of peer influence within different instructional groups on physical health outcomes. Child-initiated instruction is characterized by high frequency of play which might provide more opportunities to learn from peers. Skills gained from early peer interactions might continue beyond preschool and therefore might be linked to later obesity.

Despite the many contributions, this study also has limitations. First, the instructional approaches were reported retrospectively by Head Teachers seven years postprogram. It is possible reports did not fully represent instructional experiences because there was no direct observation. Instructional leaders who provided these reports led the program in their respective sites for years (many remained in their positions) and were validated by program evaluators. Additionally, reports met standards of inter-rater reliability, which reduced the likelihood of coder error. These findings should still be interpreted with caution. Second, we do not have teaching experience data, which might contribute to instructional quality. Based on program evaluations at the time, a substantial percentage of teachers had many years of experience. Third, we were unable to include classroom-level covariates in this study as they would be confounded with instruction, but many features were held constant as part of the CPC program. It is possible additional classroom-level factors contribute to adult outcomes. Finally, this sample is primarily low-income, Black participants from urban Chicago. Therefore, generalizability beyond urban, economically disadvantaged samples is necessarily limited. However, given the educational disparities experienced by low-income, Black families it is critical to identify educational experiences that contribute to sustained gains.

## 5 Conclusion

These results of this study suggest child-initiated and teacher-directed instruction in preschool are associated with well-being in adulthood, above and beyond risk and demographic factors. The consistent associations between child-initiated instruction and adult well-being suggest this dimension might be a stronger predictor for life-course outcomes than teacher-directed instruction. This is consistent with prior work, reporting teacher-directed instruction was predictive of immediate academic outcomes, but that these associations faded over time, whereas the association between child-initiated instruction and child outcomes was maintained into early adulthood (Graue et al., 2004). Therefore, each of these instructional dimensions are distinct and play important roles in skill development at different points in the lifespan.

A balance of high frequency child-initiated and teacher-directed activities in the preschool classroom is likely important for promoting positive outcomes for children at risk, but it is only one part of the classroom. Evidence suggests it is not only the amount of time spent in child-initiated and teacher-directed activities, but also the quality of teachers’ behaviors (Goble et al., 2016, Goble and Pianta, 2017, Hamre et al., 2014). Additionally, teacher-child relationships, classroom quality, and parent involvement are critical components of promoting positive student outcomes (Broekhuizen et al., 2016, Burchinal et al., 2014, Hamre and Pianta, 2001).

Although providing high-quality early educational experiences to children experiencing poverty is a high priority, many curricula do not provide children the opportunity for choice or discovery. Rather, evidence suggests from 1998 to 2010 kindergarten curricula focus has shifted towards direct instruction and accumulation of testable skills and the amount of time in free-choice activities has declined (Bassok et al., 2016). Similarly, many preschool classrooms spend a significant proportion of the day in teacher-directed activities, to meet rising academic skill requirements for kindergarten (Chien et al., 2010). Whereas, Goble et al. (2016) reported more time was spent in child-managed (61%) compared to teacher-directed contexts (39%) on average, with substantial individual child-initiated variability (37–92%). Although teacher-directed instruction is valuable, our findings suggest engagement in child-initiated activities in preschool is particularly important for indicators of adult well-being. Our findings add to the body of literature showing child-initiated activities in preschool are likely contributing to sustained gains from early childhood educational experiences throughout the life course. Therefore, early education efforts to blend child-initiated and teacher-directed instructional approaches will afford at-risk children the best opportunity to build important skills that can be carried into adulthood.

## Declaration of Competing Interest

The authors declare that they have no known competing financial interests or personal relationships that could have appeared to influence the work reported in this paper.
