# Supplementary material for: Preschool instructional approaches and age 35 health and well-being
Source: Prev Med Rep. 2021 Jul 19;23:101498. doi: 10.1016/j.pmedr.2021.101498 (PMC8326420; doi:10.1016/j.pmedr.2021.101498)
Supplement: Supplementary Data 1 [file mmc1.docx]

**Appendix A.** Description of Instructional Variables

Given the local flexibility in curriculum design and implementation, centers varied in their instructional philosophy and activities. Consistent with the history of the early education field, instructional philosophies have been oriented around teacher-directed and child-initiated approaches (Bowman, Donovan, & Burns, 2001). The former is highlighted by whole-class, teacher-led instruction that typically focuses on skill acquisition in pre-reading and math while the latter is more developmental and wholistic, whereby small-group and individualized activities are common and children make choices based on interests and the availability materials in the classroom (e.g., books, blocks, writing and painting supplies). Although hybrid approaches are possible, the philosophies present in the 1960s to 1980s often were distinct and not fully integrated in routine practice.

As a consequence of the natural variability in instructional approaches and the two dominant instructional philosophies present in the 1980s, CLS investigators in the early 1990s asked Head Teachers (or former Head Teachers) of each of the centers to complete a brief retrospective survey documenting the instructional approach/philosophy used in the 1983-1985 preschool period analyzed in this study (Hagemann, 1984; Mavrogenes & Oberman, 1987; Reynolds, 2000). Because all classrooms in each center followed the same model, variability occurred across and not within centers. The curricula used in the center was also reported by Head Teachers and verified by the evaluators who observed the use of these materials and were familiar with the philosophies of each center (Hagemann, 1984; Graue et al., 2004; Mavrogenes & Oberman, 1987; Reynolds, 2000). The items were chosen to reflect the orthodoxies of the time and included 12 items on a 3-point scale (low to high) covering the preschool program including the extent to which the following were implemented: formal reading instruction, basic skills approach, large group activities, small group activities, field trips, child activities (choice), teacher-directed activities, and learning (interest) centers. In addition to the Likert items described in text, teachers were asked two open response questions: 1) “Please briefly describe the teaching philosophy of the center: (e.g., direct instruction, traditional, basic skills, etc.)” and 2) “Please list the most frequently used (no more than 5) instructional materials for the classroom: (i.e., specific names of readers, workbooks, etc.).” Some of the common philosophies noted an emphasis on direct instruction, hands on activities, and/or child-centered classroom orientation. For the instructional materials open response, multiple teachers mentioned DISTAR, which is a teacher-directed curriculum. Consistently high endorsement of either teacher-directed or child-initiated dimension defined the center classification. Both Likert and open response items were used to allocate programs as high or low on teacher-directed and child-initiated, but the Likert items took precedent.

Based on the responses to survey, which were verified against program evaluator experiences in working with the centers during the years of preschool implementation (and after), centers were classified as relatively high or low on (a) teacher-directed and (b) child-initiated instructional approaches, with centers following an approach and philosophy high in both teacher-directed and child-initiated instruction, low in both, or a combination of one or the other (high in teacher-directed and low in child-initiated and the reverse). This is the 4-group classification used in the study, and this approach has a long history of analysis during the K-12 and early adult years of the project (Clements, Reynolds, & Hickey, 2004; Graue et al., 2004; Hayakawa & Reynolds, 2014; Reynolds, 2000).

References

Bowman, B. T., Donovan, M. S., & Burns, M. S. (Eds.). (2001). *Eager to learn: Education our preschoolers*. National Research Council. Washington, DC: National Academy Press.

Clements, M. A., Reynolds, A. J., & Hickey, E. (2004). Site-level predictors of school and social competence in the Chicago Child-Parent Centers. *Early Childhood Research Quarterly, 19*, 273-296.

Hagemann, M. (1984). *Chicago’s early childhood programs*. Chicago: Department of Research and Evaluation, Bureau of ECIA Program Evaluation, Chicago Public School District.

Hayakawa, M., & Reynolds, A. J. (2014). Preschool instruction approach and adult well-being. In A. J. Reynolds, A. J. Rolnick, & J. A. Temple. (Eds.), *Health and education in early childhood: Predictors, interventions, and policies* (pp. 293-316). New York: Cambridge University Press.

Mavrogenes, N. A., & Oberman, G. L. (1987, June). *Chicago Public Schools’ All-day kindergarten program: Final evaluation report, Fiscal 1986*. Chicago: Department of Research and Evaluation, Bureau of ECIA Program Evaluation for the Education Consolidation and Improvement Act, Chapter 2.

Reynolds, A. J. (2000). Success in early intervention: The Chicago Child-Parent Centers. Lincoln, NE: University of Nebraska Press.

| **Appendix B.** Descriptions for All Model Covariate Variables | |
| --- | --- |
| *Variable Name* | *Variable Description* |
| Female | Female participants coded as "1" and male participants coded as “0." The data were obtained through school records. |
| Black | Participants were coded as “1” if they were Black and “0” if they were Hispanic. This information was obtained through school records. |
| Residing in high-poverty neighborhood | The mean of five different values from the 1980 census were used to calculate the residing in high-poverty neighborhood index. The five neighborhood values obtained through the census tract and birth records included: percentage of individuals below poverty level, percentage of households with public assistance, percent of unemployed population 16 years or older, percentage of female householders with and without children in a household, and percentage of people who are 18 years or younger. |
| School-level poverty | The data were obtained from the Chicago Public School Student Information System (CPSSIS). Poverty rate values (ranging from 0% to 100%) were assigned to all participants based on their school attendance region in kindergarten. These continuous poverty rate values were then used to create a dummy variable. In the dummy coded school attendance in a high-poverty neighborhood variable, participants were coded a "1" when the percentage of children within a school district whose families live below the U.S. poverty threshold exceeds 59% and a “0” otherwise. |
| Child eligible for subsidized meals | Participants that were eligible to receive subsidized school lunch (family income < 130% federal poverty level), were coded a "1" and those who were not were coded a "0". Age-appropriate data for free lunch eligibility does not exist before the child is four years of age. Because of this, a parent survey item asking whether or not their child was eligible for free lunch in second grade was used. In cases where this item was missing, the same question asked in the child’s fourth grade data was used. In cases of remaining missing data, this variable was filled in using the participation in public aid variable (AFDC/TANF). 53 of 989 cases were filled in using the participation in public aid variable. |
| Participation in Public Aid | If the participant's family participated in public assistance programs, including Temporary Assistance to Needy Families (TANF) or Aid to Families with Dependent Children (AFDC) at the first data collection point they were coded "1" if they did not they were coded "0." In order to estimate family AFDC receipt before the child was four years old, all families receiving AFDC benefits on February 1, 1989 were considered to likely have received AFDC benefits before their child’s fourth birthday. 57 of 989 cases were imputed using the Expectation Maximization (EM) algorithm in LISREL. |
| Mother Did Not Completed High School | Mothers of participants who had not completed high school by the participant's fourth birthday were coded as "1." Mothers of the participants who had completed high school at the time of the child's birth were coded a "0." Data were obtained via parent surveys, IDPH, ILPARD, and student reports. 35 of 989 cases were imputed using the Expectation Maximization (EM) algorithm in LISREL. |
| Mother Under Age 18 at Child Birth | If the mother of the participant under the age of 18 at the time of the participant’s birth they were coded "1." If the mother was 18 or older at the time of the participant's birth they were coded "0." Data were obtained from the Illinois Department of Public Health (IDPH), the Illinois Public Assistance Research Database (ILPARD), parent reports, and student reports. The birth record data was used first, then supplemented by student, parent and administrative data. 22 of 989 cases were imputed using the Expectation Maximization (EM) algorithm in LISREL. |
| Mother not Employed Full/Part Time | Participant's with a mother who was not employed full or part time by the time their child was three years old were coded "1." If the mother was employed the participant was coded “0." The data were obtained via parent surveys and records from CPSSIS and ILPARD. Participant's whose mother reported no monthly income on their child’s free lunch application, or reported no work history when applying for public aid, were considered to not be employed full or part time. To approximate maternal employment prior to the child's fourth birthday, an item on the 11^th^ grade parent survey was used. This item asked whether or not the mother was employed when their child was in preschool. The remaining missing data were filled in with parent’s self-reported employment status when the child was in fourth grade, if this was missing then data from ILPARD were used. 115 of 989 cases were imputed using the Expectation Maximization (EM) algorithm in LISREL. |
| Single Parent Family Status | Participants in a single parent household at the time of their birth were coded “1”. Those that were not in a single parent household at birth were coded “0”. Data were obtained via birth records from IDPH, ILPARD, parent, and student reports. 30 of 989 cases were imputed using the Expectation Maximization (EM) algorithm in LISREL. |
| **Appendix B** cont'd. |  |
| Four or more children in the household | The participant was coded as “1” if there were four or more children in the household. If there were not, they were coded as “0”. Data were derived from parent and student surveys, IDPH, ILPARD, and the Chicago Public School Student Information System (CPSSIS). 30 of 989 cases were imputed using the Expectation Maximization (EM) algorithm in LISREL. |
| Child Welfare Services | Participants who had any history of child protective services due to allegations of abuse or neglect from birth to 18 years of age were coded "1" and if there was not they were coded "0." Data were obtained from the Cook County Juvenile Court (CCJC), CPSSIS, Department of Children and Family Services (DCFS), and Child and Youth Centered Information System (CYCIS). |
| Frequent Family conflict | Participants were coded as a “1” if frequent family conflict from birth to age five was reported on the adult survey response. Otherwise, they received a code of “0.” Prior to imputation the sample size from the original 989 sample was 988. 238 cases were imputed using multiple imputation. The variables used to impute included: mother under 18 years old at child birth, four or more children in the household, gender, residing in a high-poverty neighborhood, low birthweight, participation in public aid, child eligible for subsidized meals, single parent family status, mother did not complete high school, and mother not employed full or part time |
| Family financial problems | Participants were coded as a “1” if family financial problems from birth to age five were reported on an adult survey item. Otherwise, they received a code of “0.” Prior to imputation the sample size from the original 989 sample was 988. 238 cases were imputed using multiple imputation. The variables used to impute included: mother under 18 years old at child birth, four or more children in the household, gender, residing in a high-poverty neighborhood, low birthweight, participation in public aid, child eligible for subsidized meals, single parent family status, mother did not complete high school, and mother not employed full or part time |
| Substance abuse parent | Participants were coded as a “1” if parental substance abuse from birth to age five was endorsed in an item on the adult survey. Otherwise, they received a code of “0.” Prior to imputation the sample size from the original 989 sample was 988. 238 cases were imputed using multiple imputation. The variables used to impute included: mother under 18 years old at child birth, four or more children in the household, gender, residing in a high-poverty neighborhood, low birthweight, participation in public aid, child eligible for subsidized meals, single parent family status, mother did not complete high school, and mother not employed full or part time |
| Low birthweight (<2,500 grams) | Birthweights were drawn from birth certificates. Participants with a birthweight <2,500 grams (5 pounds, 8 ounces) were coded "1." If not, they were coded a "0." Data were obtained in 2002 from birth records from the Illinois Department of Public Health for students that could be matched on name and date of birth. 44 of 989 participants were imputed. |
| Participation in CPC program beyond preschool | Participants that attended the CPC (Child-Parent Center) program between ages 6 and 9 years, they received a “1.” All other participants were coded as “0”. This information was taken from school records. |
| Number of years in CPC preschool | Number of years of CPC preschool ranged from 0 to 2 in the original 1539 sample. In this sample, all children had at least one year of CPC preschool. This information was taken from school records. |
| Missing Risk Variable | Participants missing any of the following risk variables: mother under 18 years old at child birth, mother did not complete high school, single parent family status, four or more children in the household**,** participation in public aid, child eligible for subsidized meals, mother not employed full or part time, or residing in high-poverty neighborhood, between the ages of birth and three were coded "1." If not, they received a code of "0." |
| Risk Index for ages 0 to 3 | This variable was only included as a covariate in select robustness models. Each participant’s risk index was computed using 8 dichotomous variables: mother under 18 years old at child birth, mother did not complete high school, single parent family status, four or more children in the household**,** participation in public aid, child eligible for subsidized meals, mother not employed full or part time, or residing in high-poverty neighborhood, between the ages of birth and three. Values ranged from 0 to 8, with higher numbers indicating greater risk. Data were complete for this variable following imputation using the EM algorithm in LISERL. |

| **Appendix C.** Correlations Between All Outcomes and Predictor Variables | | | | | | | | | | | | | | | | |
| --- | --- | --- | --- | --- | --- | --- | --- | --- | --- | --- | --- | --- | --- | --- | --- | --- |
|  | Variables | 1 | | 2 | 3 | 4 | 5 | 6 | 7 | 8 | 9 | 10 | 11 | 12 | 13 | 14 |
| 1 | HT + HC | - | |  |  |  |  |  |  |  |  |  |  |  |  |  |
| 2 | HT + LC | -.209^**^ | | - |  |  |  |  |  |  |  |  |  |  |  |  |
| 3 | LT + HC | -.611^**^ | | -.199^**^ | - |  |  |  |  |  |  |  |  |  |  |  |
| 4 | LT + LC | -.373^**^ | | -.121^**^ | -.354^**^ | - |  |  |  |  |  |  |  |  |  |  |
| 5 | Livable Income | 0.053 | | -0.053 | .073^*^ | -.125^**^ | - |  |  |  |  |  |  |  |  |  |
| 6 | Associate's degree | 0.013 | | -.065^*^ | 0.025 | -0.006 | .272^**^ | - |  |  |  |  |  |  |  |  |
| 7 | Bachelor's degree | .067^*^ | | -0.056 | -0.015 | -0.031 | .283^**^ | .811^**^ | - |  |  |  |  |  |  |  |
| 8 | Felony arrest | -.067^*^ | | -0.005 | -0.036 | .135^**^ | -.276^**^ | -.254^**^ | -.220^**^ | - |  |  |  |  |  |  |
| 9 | Jail or incarceration | -.081^*^ | | 0.052 | -0.015 | .089^**^ | -.265^**^ | -.196^**^ | -.174^**^ | .595^**^ | - |  |  |  |  |  |
| 10 | Conviction | -0.036 | | -0.024 | -0.043 | .114^**^ | -.269^**^ | -.247^**^ | -.228^**^ | .707^**^ | .578^**^ | - |  |  |  |  |
| 11 | Current Smoking | 0.031 | | -0.036 | -0.008 | -0.005 | -.139^**^ | -.168^**^ | -.181^**^ | .218^**^ | .184^**^ | .191^**^ | - |  |  |  |
| 12 | Obesity (BMI ≥ 30) | 0.028 | | -0.039 | 0.010 | -0.023 | -0.002 | -0.017 | -0.050 | -0.033 | -0.073 | -0.016 | 0.013 | - |  |  |
| 13 | Female child | 0.002 | | 0.036 | 0.008 | -0.035 | 0.033 | .172^**^ | .108^**^ | -.338^**^ | -.414^**^ | -.355^**^ | -.222^**^ | 0.064 | - |  |
| 14 | Black | .201^**^ | | 0.057 | -.190^**^ | -0.053 | -0.041 | -.079^*^ | -0.047 | 0.030 | 0.039 | .075^*^ | 0.069 | 0.019 | 0.002 | - |
| 15 | School-level poverty | -.112^**^ | | .140^**^ | -.156^**^ | .250^**^ | -0.063 | .075^*^ | 0.051 | -0.033 | -0.029 | -0.005 | -0.031 | -0.074 | 0.046 | -.122^**^ |
| 16 | Residing in high-poverty neighborhood | .099^**^ | | -0.027 | -0.034 | -.066^*^ | -.072^*^ | -.073^*^ | -.097^**^ | 0.024 | 0.042 | 0.004 | 0.049 | -0.023 | 0.005 | .129^**^ |
| 17 | Child eligible for subsidized meals | -0.022 | | 0.022 | 0.001 | 0.013 | -.069^*^ | -0.062 | -.076^*^ | 0.008 | 0.013 | -0.003 | 0.007 | 0.003 | 0.049 | 0.039 |
| 18 | Participation in Public Aid | 0.021 | | 0.062 | -0.052 | 0.000 | -.136^**^ | -.092^**^ | -.116^**^ | 0.017 | -0.005 | 0.046 | .076^*^ | -0.010 | .064^*^ | .157^**^ |
| 19 | Mother Did Not Completed High School | -0.030 | | 0.049 | -0.021 | 0.033 | -.084^*^ | -.118^**^ | -.117^**^ | .076^*^ | 0.044 | .095^**^ | 0.023 | -0.020 | -0.051 | -.104^**^ |
| 20 | Mother Under Age 18 at Child Birth | 0.016 | | 0.002 | -0.009 | -0.010 | 0.024 | -0.061 | -0.033 | 0.043 | 0.019 | 0.031 | 0.006 | -0.016 | -0.021 | .077^*^ |
| 21 | Mother not Employed Full/Part Time | 0.024 | | 0.014 | -0.029 | -0.003 | -.116^**^ | -.078^*^ | -.086^**^ | -0.007 | -0.031 | 0.032 | 0.042 | 0.042 | .071^*^ | .095^**^ |
| 22 | Single Parent Family Status | .083^**^ | | 0.026 | -.072^*^ | -0.032 | -.102^**^ | -.097^**^ | -.113^**^ | 0.014 | 0.000 | -0.002 | 0.013 | -0.008 | 0.044 | .131^**^ |
| 23 | Four or more children in the household | 0.041 | | -0.035 | -0.034 | 0.014 | -0.039 | -0.040 | -0.043 | 0.023 | 0.013 | 0.016 | .084^*^ | 0.063 | 0.001 | -0.037 |
| 24 | Child Welfare Services | -0.049 | | 0.024 | 0.056 | -0.023 | -0.064 | -.068^*^ | -.069^*^ | 0.017 | 0.007 | 0.021 | -0.015 | 0.021 | 0.011 | -0.017 |
| 25 | Frequent Family conflict | -0.044 | | 0.061 | 0.040 | -0.033 | -0.003 | -0.030 | -0.006 | 0.035 | 0.057 | 0.053 | 0.063 | -0.062 | -0.044 | 0.001 |
| 26 | Family Financial Problems | -0.003 | | 0.009 | -0.014 | 0.017 | -0.042 | 0.019 | 0.020 | 0.042 | .098^**^ | 0.036 | 0.045 | -0.035 | -.081^*^ | -0.014 |
| 27 | Substance abuse parent | 0.026 | | -0.014 | 0.006 | -0.032 | -0.013 | 0.030 | 0.024 | 0.049 | .085^**^ | 0.046 | .104^**^ | -0.071 | -0.028 | 0.001 |
| 28 | Low birthweight (<2,500 grams) | -0.035 | | 0.002 | 0.050 | -0.019 | -0.034 | -0.043 | -0.030 | -0.006 | -0.011 | 0.003 | -0.047 | -0.030 | 0.039 | .098^**^ |
| 29 | Participation in CPC program beyond preschool | | -0.021 | -0.014 | 0.054 | -0.033 | 0.049 | 0.036 | .070^*^ | 0.005 | 0.018 | -0.001 | -0.014 | -0.038 | 0.024 | -0.010 |
| 30 | Number of years in CPC preschool | | 0.033 | 0.05 | -0.042 | -0.021 | -0.03 | -0.027 | -0.01 | -.063^*^ | -0.054 | -0.03 | -0.019 | 0.039 | 0.058 | .085^**^ |
| 31 | Missing Risk Variable | | .069^*^ | -0.027 | -0.009 | -0.059 | 0.012 | 0.031 | 0.019 | -0.046 | 0.016 | -0.059 | 0.042 | -0.04 | -0.035 | -0.026 |

| **Appendix C**. cont'd. | | | | | | | | | | | | | | | | | | | | | | |  | |  |
| --- | --- | --- | --- | --- | --- | --- | --- | --- | --- | --- | --- | --- | --- | --- | --- | --- | --- | --- | --- | --- | --- | --- | --- | --- | --- |
|  | 15 | 16 | 17 | | 18 | 19 | 20 | | 21 | 22 | 23 | | 24 | 25 | 26 | | 27 | 28 | 28 | | 30 | 31 | |  |  |
| 1 |  |  |  | |  |  |  | |  |  |  | |  |  |  | |  |  |  | |  |  | |  |  |
| 2 |  |  |  | |  |  |  | |  |  |  | |  |  |  | |  |  |  | |  |  | |  |  |
| 3 |  |  |  | |  |  |  | |  |  |  | |  |  |  | |  |  |  | |  |  | |  |  |
| 4 |  |  |  | |  |  |  | |  |  |  | |  |  |  | |  |  |  | |  |  | |  |  |
| 5 |  |  |  | |  |  |  | |  |  |  | |  |  |  | |  |  |  | |  |  | |  |  |
| 6 |  |  |  | |  |  |  | |  |  |  | |  |  |  | |  |  |  | |  |  | |  |  |
| 7 |  |  |  | |  |  |  | |  |  |  | |  |  |  | |  |  |  | |  |  | |  |  |
| 8 |  |  |  | |  |  |  | |  |  |  | |  |  |  | |  |  |  | |  |  | |  |  |
| 9 |  |  |  | |  |  |  | |  |  |  | |  |  |  | |  |  |  | |  |  | |  |  |
| 10 |  |  |  | |  |  |  | |  |  |  | |  |  |  | |  |  |  | |  |  | |  |  |
| 11 |  |  |  | |  |  |  | |  |  |  | |  |  |  | |  |  |  | |  |  | |  |  |
| 12 |  |  |  | |  |  |  | |  |  |  | |  |  |  | |  |  |  | |  |  | |  |  |
| 13 |  |  |  | |  |  |  | |  |  |  | |  |  |  | |  |  |  | |  |  | |  |  |
| 14 |  |  |  | |  |  |  | |  |  |  | |  |  |  | |  |  |  | |  |  | |  |  |
| 15 | - |  |  | |  |  |  | |  |  |  | |  |  |  | |  |  |  | |  |  | |  |  |
| 16 | -0.015 | - |  | |  |  |  | |  |  |  | |  |  |  | |  |  |  | |  |  | |  |  |
| 17 | 0.034 | .130^**^ | - | |  |  |  | |  |  |  | |  |  |  | |  |  |  | |  |  | |  |  |
| 18 | 0.017 | .158^**^ | .393^**^ | | - |  |  | |  |  |  | |  |  |  | |  |  |  | |  |  | |  |  |
| 19 | .071^*^ | .108^**^ | .158^**^ | | .222^**^ | - |  | |  |  |  | |  |  |  | |  |  |  | |  |  | |  |  |
| 20 | -0.017 | .106^**^ | .063^*^ | | .109^**^ | .321^**^ | - | |  |  |  | |  |  |  | |  |  |  | |  |  | |  |  |
| 21 | 0.009 | .137^**^ | .337^**^ | | .678^**^ | .192^**^ | 0.055 | | - |  |  | |  |  |  | |  |  |  | |  |  | |  |  |
| 22 | -0.020 | .143^**^ | .116^**^ | | .249^**^ | .125^**^ | .210^**^ | | .183^**^ | - |  | |  |  |  | |  |  |  | |  |  | |  |  |
| 23 | 0.035 | 0.046 | 0.052 | | -0.038 | .069^*^ | -.187^**^ | | -0.002 | -.211^**^ | - | |  |  |  | |  |  |  | |  |  | |  |  |
| 24 | -0.043 | -0.020 | 0.014 | | -0.031 | 0.060 | 0.019 | | -0.036 | 0.058 | 0.032 | | - |  |  | |  |  |  | |  |  | |  |  |
| 25 | -0.036 | -0.048 | 0.022 | | 0.033 | 0.022 | 0.003 | | 0.022 | 0.021 | -0.035 | | 0.031 | - |  | |  |  |  | |  |  | |  |  |
| 26 | -0.022 | -0.008 | 0.044 | | 0.056 | -0.044 | 0.023 | | 0.033 | -0.044 | 0.019 | | -0.027 | .308^**^ | - | |  |  |  | |  |  | |  |  |
| 27 | -0.007 | -0.050 | -0.019 | | 0.047 | -0.024 | 0.020 | | 0.019 | 0.033 | -.065^*^ | | -0.038 | .295^**^ | .274^**^ | | - |  |  | |  |  | |  |  |
| 28 | 0.001 | 0.059 | -0.009 | | .093^**^ | 0.019 | 0.037 | | .078^*^ | .085^**^ | -0.020 | | 0.030 | -0.030 | -0.008 | | -0.010 | - |  | |  |  | |  |  |
| 29 | -0.048 | -.104^**^ | -0.055 | | -0.030 | -0.038 | -0.009 | | -0.059 | 0.006 | -0.002 | | 0.032 | 0.040 | -0.021 | | -0.001 | 0.009 | - | |  |  | |  |  |
| 30 | 0.026 | .098^**^ | -0.004 | | -0.012 | -.098^**^ | -0.051 | | 0.002 | -0.062 | 0.032 | | -.078^*^ | -0.002 | 0.041 | | -0.037 | 0.011 | 0.025 | | - |  | |  |  |
| 31 | -0.044 | 0.034 | .078^*^ | | .117^**^ | -0.059 | -0.061 | | .120^**^ | 0 | -0.049 | | -0.026 | -0.04 | -0.014 | | 0.026 | -0.045 | -.129^**^ | | -0.028 | - | |  |  |
| *Note:* ** *p*<.01; * *p*<.05. | | | |  | | | |  | | | |  | | | |  | | | |  | | | | | |

| **Appendix D.** Logistic Hierarchical Regression Odds Ratio and Confidence Intervals for Models Predicting Age 35 Outcomes | | | | | | | | | | | | | | | | |  |
| --- | --- | --- | --- | --- | --- | --- | --- | --- | --- | --- | --- | --- | --- | --- | --- | --- | --- |
|  | | Livable Wage | | AA | BA | | Felony Arrests | | Jail or incarceration | | Conviction | | Smoking | | Obesity (BMI ≥ 30) |  |  |
| Model 1 (unadjusted) |  | |  | | |  | |  | |  | |  | |  | |  |  |
| HT+HC | | 1.936** (1.302, 2.878) | | 1.053 (0.65, 1.706) | 1.463 (0.829, 2.582) | | 0.456** (0.313, 0.664) | | 0.481** (0.304, 0.76) | | 0.543** (0.376, 0.786) | | 1.067 (0.683, 1.668) | | 1.23 (0.79, 1.914) |  |  |
| HT+LC | | 1.221 (0.656, 2.272) | | 0.471 (0.185, 1.201) | 0.532 (0.172, 1.642) | | 0.524* (0.283, 0.968) | | 0.978 (0.496, 1.93) | | 0.489* (0.266, 0.898) | | 0.795 (0.398, 1.592) | | 0.773 (0.389, 1.537) |  |  |
| LT+HC | | 2.146** (1.437, 3.205) | | 1.082 (0.663, 1.766) | 1.103 (0.609, 1.997) | | 0.519* (0.356, 0.756) | | 0.64* (0.41, 0.999) | | 0.565** (0.389, 0.819) | | 0.967 (0.612, 1.527) | | 1.106 (0.705, 1.736) |  |  |
| Constant | | 0.596** (0.428, 0.829) | | 0.236** (0.157, 0.353) | 0.134** (0.082, 0.22) | | 0.931 (0.688, 1.26) | | 0.333** (0.235, 0.473) | | 1.074 (0.794, 1.454) | | 0.629* (0.431, 0.916) | | 0.727 (0.501, 1.055) |  |  |
| -2 Log Likelihood | | 1137.5 | | 846.34 | 688.39 | | 1197.4 | | 876.13 | | 1256.98 | | 919.09 | | 922.49 |  |  |
| Nagelkerke *R^2^* | | 0.027 | | 0.008 | 0.012 | | 0.026 | | 0.02 | | 0.018 | | 0.002 | | 0.005 |  |  |
| Model 2 (adjusted) | |  | |  |  | |  | |  | |  | |  | |  |  |  |
| HT+HC | | 2.019** (1.315, 3.099) | | 1.278 (0.755, 2.165) | 1.879* (1.011, 3.49) | | 0.392** (0.255, 0.601) | | 0.346** (0.198, 0.603) | | 0.519** (0.341, 0.791) | | 0.945 (0.581, 1.54) | | 1.184 (0.741, 1.891) |  |  |
| HT+LC | | 1.367 (0.717, 2.607) | | 0.5 (0.19, 1.315) | 0.63 (0.197, 2.01) | | 0.573 (0.294, 1.115) | | 1.482 (0.65, 3.377) | | 0.475* (0.243, 0.927) | | 0.784 (0.377, 1.631) | | 0.801 (0.397, 1.615) |  |  |
| LT+HC | | 2.009** (1.305, 3.093) | | 1.181 (0.695, 2.006) | 1.157 (0.614, 2.179) | | 0.46** (0.299, 0.708) | | 0.529* (0.306, 0.916) | | 0.569** (0.372, 0.872) | | 1.017 (0.615, 1.682) | | 1.054 (0.653, 1.703) |  |  |
| Female child | | 1.288 (0.964, 1.72) | | 2.997** (2.023, 4.442) | 2.388** (1.531, 3.723) | | 0.213** (0.157, 0.289) | | 0.045** (0.024, 0.083) | | 0.201** (0.15, 0.27) | | 0.357** (0.256, 0.499) | | 1.252 (0.909, 1.724) |  |  |
| Black child | | 0.757 (0.413, 1.388) | | 0.607 (0.304, 1.211) | 0.56 (0.257, 1.221) | | 1.208 (0.645, 2.262) | | 1.51 (0.635, 3.587) | | 1.91* (1.013, 3.6) | | 1.776 (0.828, 3.81) | | 0.937 (0.478, 1.835) |  |  |
| School-level poverty | | 0.927 (0.647, 1.328) | | 1.905* (1.159, 3.128) | 1.934* (1.096, 3.414) | | 0.736 (0.508, 1.067) | | 0.693 (0.429, 1.121) | | 0.984 (0.684, 1.416) | | 0.969 (0.644, 1.457) | | 0.669* (0.45, 0.993) |  |  |
| Residing in high-poverty neighborhood | | 0.623 (0.253, 1.53) | | 0.6 (0.198, 1.813) | 0.368 (0.105, 1.293) | | 1.634 (0.646, 4.134) | | 3.689* (1.09, 12.481) | | 0.981 (0.399, 2.413) | | 1.622 (0.576, 4.567) | | 0.562 (0.208, 1.524) |  |  |
| Child eligible for subsidized meals | | 0.863 (0.566, 1.315) | | 0.767 (0.463, 1.271) | 0.79 (0.452, 1.379) | | 1.073 (0.688, 1.674) | | 1.327 (0.744, 2.368) | | 0.924 (0.6, 1.425) | | 0.998 (0.62, 1.604) | | 0.961 (0.611, 1.514) |  |  |
| Participation in Public Aid (AFDC) | | 0.714 (0.473, 1.079) | | 0.952 (0.564, 1.607) | 0.696 (0.382, 1.269) | | 1.095 (0.707, 1.696) | | 1.003 (0.563, 1.789) | | 1.232 (0.803, 1.892) | | 1.425 (0.89, 2.283) | | 0.822 (0.523, 1.292) |  |  |
| Mother Did Not Completed High School | | 0.733 (0.532, 1.009) | | 0.667 (0.441, 1.007) | 0.601* (0.369, 0.978) | | 1.173 (0.843, 1.633) | | 1.17 (0.759, 1.802) | | 1.372 (0.993, 1.897) | | 0.998 (0.691, 1.44) | | 0.996 (0.699, 1.42) |  |  |
| Mother Under Age 18 at Child Birth | | 1.548* (1.007, 2.379) | | 0.847 (0.47, 1.527) | 1.176 (0.605, 2.287) | | 1.2 (0.779, 1.849) | | 1.045 (0.593, 1.841) | | 1.009 (0.659, 1.545) | | 1.035 (0.632, 1.697) | | 1.022 (0.633, 1.651) |  |  |
| Mother not Employed Full/Part Time | | 0.813 (0.542,1.218) | | 0.803 (0.488, 1.321) | 0.934 (0.53, 1.644) | | 0.962 (0.626, 1.479) | | 0.768 (0.433, 1.362) | | 1.151 (0.755, 1.755) | | 1.003 (0.629, 1.598) | | 1.426 (0.913, 2.228) |  |  |
| Single Parent Family Status | | 0.674* (0.469, 0.968) | | 0.651* (0.426, 0.995) | 0.573* (0.356, 0.921) | | 1.055 (0.723, 1.539) | | 1.159 (0.705, 1.903) | | 0.891 (0.617, 1.286) | | 1.053 (0.7, 1.585) | | 1.014 (0.689, 1.492) |  |  |
| Four or more children in the household | | 0.827 (0.552, 1.24) | | 0.736 (0.434, 1.249) | 0.677 (0.361, 1.268) | | 1.266 (0.838, 1.914) | | 1.21 (0.709, 2.066) | | 1.078 (0.718, 1.618) | | 1.654* (1.053, 2.6) | | 1.351 (0.871, 2.097) |  |  |
| Child Welfare Services | | 0.501 (0.201, 1.247) | | 0.193 (0.025, 1.5) | 0 (0, Inf) | | 1.268 (0.55, 2.923) | | 0.981 (0.33, 2.922) | | 1.504 (0.658, 3.44) | | 0.854 (0.32, 2.276) | | 0.966 (0.383, 2.439) |  |  |
| Frequent Family conflict | | 1.195 (0.62, 2.303) | | 0.697 (0.287, 1.692) | 1.024 (0.4, 2.62) | | 1.045 (0.542, 2.015) | | 0.885 (0.386, 2.028) | | 1.326 (0.699, 2.514) | | 1.439 (0.715, 2.898) | | 0.622 (0.297, 1.299) |  |  |
| Family Financial Problems | | 0.725 (0.405, 1.298) | | 1.592 (0.797, 3.179) | 1.523 (0.698, 3.327) | | 0.982 (0.543, 1.774) | | 1.512 (0.745, 3.069) | | 0.89 (0.496, 1.594) | | 0.838 (0.444, 1.58) | | 0.936 (0.5, 1.751) |  |  |
| Substance abuse parent | | 0.799 (0.389, 1.641) | | 1.272 (0.538, 3.006) | 0.974 (0.355, 2.674) | | 1.901 (0.91, 3.972) | | 3.241* (1.309, 8.023) | | 1.659 (0.792, 3.478) | | 2.482* (1.129, 5.46) | | 0.559 (0.238, 1.31) |  |  |
| Low birthweight (<2,500 grams) | | 0.86 (0.544, 1.36) | | 0.792 (0.432, 1.452) | 0.964 (0.483, 1.926) | | 1.008 (0.63, 1.612) | | 1.022 (0.544, 1.921) | | 1.041 (0.662, 1.638) | | 0.748 (0.447, 1.252) | | 0.811 (0.497, 1.323) |  |  |
| Participation in CPC program beyond preschool | | 1.204 (0.881, 1.644) | | 1.289 (0.861, 1.929) | 1.656* (1.019, 2.691) | | 1.013 (0.734, 1.397) | | 1.25 (0.817, 1.912) | | 1.004 (0.733, 1.376) | | 1.022 (0.713, 1.465) | | 0.849 (0.601, 1.2) |  |  |
| Number of years of CPC preschool | | 0.873 (0.653, 1.169) | | 0.771 (0.533, 1.114) | 0.797 (0.521, 1.219) | | 0.811 (0.602, 1.094) | | 0.74 (0.5, 1.095) | | 0.942 (0.703, 1.262) | | 0.95 (0.682, 1.323) | | 1.213 (0.882, 1.668) |  |  |
| Missing Risk Variable | | 1.259 (0.777, 2.038) | | 1.334 (0.737, 2.411) | 1.461 (0.745, 2.864) | | 0.603 (0.363, 1.003) | | 1.235 (0.671, 2.272) | | 0.594* (0.365, 0.967) | | 1.081 (0.627, 1.866) | | 0.771 (0.448, 1.327) |  |  |
| Constant | | 2.403 (0.895, 6.455) | | 0.404 (0.122, 1.337) | 0.299 (0.076, 1.166) | | 1.838 (0.661, 5.115) | | 0.42 (0.106, 1.655) | | 1.12 (0.408, 3.073) | | 0.398 (0.121, 1.302) | | 0.992 (0.329, 2.99) |  |  |
| -2 Log Likelihood | | 1091.1 | | 770.59 | 620.38 | | 1066.5 | | 664.05 | | 1108.6 | | 857.34 | | 901.8 |  |  |
| Nagelkerke *R^2^* | | 0.098 | | 0.141 | 0.148 | | 0.201 | | 0.346 | | 0.212 | | 0.118 | | 0.045 |  |  |
| *N* | | 833 | | 869 | 869 | | 945 | | 945 | | 945 | | 690 | | 673 |  |  |
| *Note:* ** *p*<.01; * *p*<.05. HT+HC = High teacher-directed and high child-initiated, HT+LC = High teacher-directed and low child-initiated, LT+HC = Low teacher-directed and high child-initiated, LT+LC = Low teacher-directed and low child-initiated. | | | | | | | | | | | | | | | | | |

| **Appendix E.** Logistic Hierarchical Regression odds ratio and confidence intervals for Models Predicting Age 35 Outcomes using Inverse Propensity Score Weighting | | | | | |
| --- | --- | --- | --- | --- | --- |
|  | Income | AA | BA | Smoking | Obesity (BMI ≥ 30) |
| Model 1 (unadjusted) |  |  |  |  |  |
| HT + HC | 1.99** (1.37, 2.89) | 1.09 (0.68, 1.73) | 1.55 (0.89, 2.7w) | 1.08 (0.74, 1.57) | 1.20 (0.83, 1.74) |
| HT + LC | 1.18 (0.65, 2.13) | 0.46 (0.18, 1.17) | 0.55 (0.18, 1.70) | 0.73 (0.40, 1.33) | 0.85 (0.47, 1.51) |
| LT + HC | 2.09** (1.43, 3.05) | 1.19 (0.74, 1.89) | 1.11 (0.62, 1.99) | 0.94 (0.64, 1.37) | 1.16 (0.79, 1.69) |
| Constant | 0.56** (0.41, 0.76) | 0.23** (0.16, 0.34) | 0.12** (0.08, 0.20) | 0.67* (0.49, 0.92) | 0.71* (0.52, 0.98) |
| -2 Log Likelihood | 1304.1 | 943.47 | 738.72 | 1267.2 | 1293.2 |
| Nagelkerke *R^2^* | 0.03 | 0.01 | 0.01 | 0 | 0 |
| Model 2 (adjusted) |  |  |  |  |  |
| HT + HC | 1.98** (1.35, 2.90) | 1.09 (0.67, 1.78) | 1.49 (0.84, 2.66) | 1.03 (0.70, 1.52) | 1.19 (0.82, 1.73) |
| HT + LC | 1.25 (0.69, 2.29) | 0.47 (0.18, 1.22) | 0.57 (0.18, 1.78) | 0.69 (0.37, 1.28) | 0.83 (0.46, 1.49) |
| LT + HC | 1.94** (1.32, 2.85) | 0.98 (0.60, 1.60) | .94 (0.52, 1.72) | 1.04 (0.70, 1.55) | 1.16 (0.79, 1.69) |
| Female child | 1.10 (0.84, 1.42) | 2.84** (1.98, 4.07) | 2.03** (1.35, 3.05) | 0.38** (0.29, 0.50) | 1.33* (1.02, 1.72) |
| Black child | 0.74 (0.44, 1.24) | 0.37** (0.21, 0.64) | 0.63 (0.32, 1.26) | 2.16* (1.21, 3.85) | 1.16 (0.70, 1.93) |
| Low birthweight (<2,500 grams) | 0.78 (0.51, 1.18) | 0.78 (0.43, 1.40) | 0.90 (0.46, 1.75) | 0.70 (0.45, 1.10) | 0.81 (0.53, 1.23) |
| Risk Index from age 0-3 | 0.83** (0.76, 0.90) | 0.82** (0.74, 0.90) | 0.77** (0.69, 0.87) | 1.12* (1.03, 1.21) | 1.00 (0.92, 1.08) |
| Constant | 1.74 (0.88, 3.41) | 0.79 (0.36,1.73) | 0.40 (0.16, 1.02) | 0.34** (0.16, 0.71) | 0.55 (0.28, 1.07) |
| -2 Log Likelihood | 1278.2 | 881.26 | 706.69 | 1203.5 | 1287.6 |
| Nagelkerke *R^2^* | 0.06 | 0.11 | 0.08 | 0.10 | 0.01 |
| *N* | 871 | 904 | 904 | 722 | 704 |

| **Appendix F.** Logistic Hierarchical Regression Odds Ratio and Confidence Intervals for Models Predicting Age 35 Outcomes Using Inverse Propensity Score Weighting and Larger Set of Risk Covariates | | | | | |
| --- | --- | --- | --- | --- | --- |
|  | Income | AA | BA | Smoking | Obesity (BMI ≥ 30) |
| Model 1 (unadjusted) | |  |  |  |  |
| HT + HC | 1.963** (1.34, 2.875) | 1.05 (0.652, 1.691) | 1.448 (0.822, 2.551) | 1.038 (0.707, 1.525) | 1.242 (0.849, 1.817) |
| HT + LC | 1.237 (0.679, 2.255) | 0.467 (0.184, 1.187) | 0.538 (0.175, 1.659) | 0.755 (0.412, 1.383) | 0.773 (0.426, 1.401) |
| LT + HC | 2.141** (1.455, 3.151) | 1.157 (0.717, 1.865) | 1.052 (0.583, 1.898) | 0.89 (0.601, 1.318) | 1.124 (0.763, 1.655) |
| Constant | 0.562** (0.408, 0.774) | 0.233** (0.156, 0.347) | 0.13** (0.08, 0.212) | 0.682* (0.494, 0.941) | 0.73 (0.531, 1.003) |
| -2 Log Likelihood | 1242.6 | 897.57 | 704.64 | 1202.1 | 1231.4 |
| Nagelkerke *R^2^* | 0.03 | 0.009 | 0.01 | 0.003 | 0.005 |
| Model 2 (adjusted) | |  |  |  |  |
| HT + HC | 2.042** (1.352, 3.086) | 1.305 (0.771, 2.208) | 1.803 (0.974, 3.339) | 0.917 (0.602, 1.396) | 1.205 (0.806, 1.802) |
| HT + LC | 1.384 (0.741, 2.585) | 0.505 (0.192, 1.324) | 0.619 (0.195, 1.969) | 0.743 (0.392, 1.409) | 0.802 (0.437, 1.472) |
| LT + HC | 2.001** (1.32, 3.033) | 1.152 (0.681, 1.949) | 1.071 (0.571, 2.011) | 0.93 (0.602, 1.436) | 1.075 (0.713, 1.622) |
| Female child | 1.223 (0.927, 1.613) | 3.039** (2.066, 4.471) | 2.209** (1.425, 3.423) | 0.347** (0.259, 0.465) | 1.264 (0.959, 1.666) |
| Black Child | 0.738 (0.414, 1.318) | 0.411** (0.218, 0.773) | 0.579 (0.271, 1.239) | 2.003* (1.046, 3.834) | 0.904 (0.514, 1.587) |
| School-level poverty | 0.883 (0.625, 1.246) | 1.811* (1.104, 2.969) | 1.799* (1.02, 3.171) | 1.003 (0.697, 1.442) | 0.674* (0.476, 0.954) |
| Residing in high-poverty neighborhood | 0.619 (0.261, 1.469) | 0.545 (0.18, 1.65) | 0.376 (0.106, 1.332) | 1.681 (0.68, 4.156) | 0.533 (0.225, 1.265) |
| Child eligible for subsidized meals | 0.841 (0.558, 1.266) | 0.776 (0.468, 1.287) | 0.788 (0.452, 1.375) | 1.031 (0.675, 1.575) | 0.923 (0.619, 1.376) |
| Participation in Public Aid (AFDC) | 0.718 (0.482, 1.07) | 1.07 (0.641, 1.786) | 0.702 (0.389, 1.265) | 1.385 (0.909, 2.109) | 0.807 (0.541, 1.203) |
| Mother Did Not Completed High School | 0.736 (0.541, 1.001) | 0.631* (0.422, 0.943) | 0.543* (0.336, 0.877) | 0.955 (0.692, 1.318) | 1.043 (0.767, 1.418) |
| Mother Under Age 18 at Child Birth | 1.509* (1.002, 2.272) | 0.887 (0.493, 1.598) | 1.263 (0.649, 2.457) | 1.08 (0.708, 1.648) | 1.038 (0.692, 1.559) |
| Mother not Employed Full/Part Time | 0.817 (0.551, 1.209) | 0.761 (0.464, 1.247) | 0.935 (0.533, 1.639) | 1.005(0.664,1.522) | 1.473 (0.993, 2.184) |
| Single Parent Family Status | 0.656* (0.464, 0.928) | 0.606* (0.401, 0.915) | 0.611* (0.382, 0.977) | 1.113(0.775,1.597) | 0.999 (0.713, 1.401) |
| Four or more children in the household | 0.819 (0.554, 1.21) | 0.678 (0.401, 1.147) | 0.718 (0.386, 1.337) | 1.671*(1.126,2.479) | 1.38 (0.946, 2.011) |
| Child Welfare Services | 0.494 (0.207, 1.183) | 0.181 (0.023, 1.425) | 0 (0, Inf) | 0.887 (0.387, 2.03) | 0.931 (0.425, 2.037) |
| Frequent Family conflict | 1.141 (0.61, 2.135) | 0.7 (0.286, 1.712) | 1.069(0.419,2.731) | 1.435(0.766,2.686) | 0.635 (0.334, 1.206) |
| Family Financial Problems | 0.731 (0.417, 1.281) | 1.596 (0.798, 3.19) | 1.57 (0.721, 3.42) | 0.886 (0.506, 1.554) | 0.983 (0.567, 1.705) |
| Substance abuse parent | 0.843 (0.415, 1.71) | 1.256 (0.532, 2.969) | 1.044 (0.383, 2.842) | 2.474* (1.219, 5.023) | 0.595 (0.285, 1.24) |
| Low birthweight (<2,500 grams) | 0.782 (0.507, 1.205) | 0.826 (0.451, 1.51) | 1.008 (0.506, 2.007) | 0.761 (0.481, 1.204) | 0.813 (0.529, 1.251) |
| Participation in CPC program beyond preschool | 1.174 (0.871, 1.584) | 1.107 (0.752, 1.631) | 1.579 (0.979, 2.546) | 1.061 (0.776, 1.451) | 0.861 (0.64, 1.159) |
| Number of years of CPC preschool | 0.867 (0.656, 1.146) | 0.746 (0.517, 1.074) | 0.798 (0.521, 1.221) | 0.915 (0.684, 1.223) | 1.214 (0.921, 1.6) |
| Missing Risk Variable | 1.284 (0.822, 2.006) | 1.172 (0.695, 1.975) | 0.887 (0.464, 1.696) | 1.057 (0.662, 1.686) | 0.797 (0.505, 1.256) |
| Constant | 2.684* (1.042, 6.916) | 0.747 (0.242, 2.31) | 0.33 (0.089, 1.226) | 0.366 (0.131,1.022) | 1.03 (0.4, 2.65) |
| -2 Log Likelihood | 1191.3 | 807.26 | 640.6 | 1117.2 | 1204.2 |
| Nagelkerke *R^2^* | 0.099 | 0.158 | 0.139 | 0.138 | 0.053 |
| *N* | 833 | 869 | 869 | 690 | 673 |

*Note:* ** *p*<.01; * *p*<.05.

| **Appendix G.**  Abbreviated propensity model used to estimate predicted probability of being in the sample. | | | | | | |
| --- | --- | --- | --- | --- | --- | --- |
|  | Income | | | Educational Attainment  (AA or BA degree) | | |
| Variables | Odds Ratio | *p*-value | | | Odds Ratio | *p*-value |
| Black | 1.886 | 0.286 | | | 15.649 | 0.015 |
| Child Welfare Services | 0.652 | 0.503 | | | 0.023 | 0.034 |
| Child eligible for subsidized meals ages 0-3 | 0.371 | 0.066 | | | 4.87 | 0.169 |
| Frequent Family Conflict | 1.22 | 0.19 | | | 6.136 | 0.001 |
| Social Emotional Maturity Grades 1-3 | 1.097 | 0.046 | | | 0.926 | 0.514 |
| Participant dropped out of high school at or before age 16 | 0.424 | 0.02 | | | 3.277 | 0.27 |
| Chi-squared | 175.869 | | 235.414 | | | |
| Nagelkerke *R^2^* | 0.368 | | 0.734 | | | |
| *N* | 946 | | 945 | | | |
| *Note*. Selected predictors (out of 54 total) are displayed here along with the fit model and fit statistics for the full model. 43 participants had passed away as of November 2012 and were not included in the model. | | | | | | |
